# Hepatitis C in Eastern Europe and Central Asia: a survey of epidemiology, treatment access and civil society activity in eleven countries

**DOI:** 10.1186/s41124-017-0026-z

**Published:** 2017-06-13

**Authors:** Ludmila Maistat, Natalija Kravchenko, Amulya Reddy

**Affiliations:** 1 Alliance for Public Health, Kiev, Ukraine; 2 London, UK

**Keywords:** Hepatitis C, Civil society, Access to treatment, Direct-acting antivirals, Treatment, Vulnerable groups, Eastern Europe and Central Asia

## Abstract

**Introduction:**

The 16 countries of the Eastern Europe and Central Asia (EECA) region are home to 6.6 million people in need of treatment for chronic hepatitis C virus (HCV) infection. Because of transformational change in HCV treatment, global efforts to address HCV are accelerating. Given its large regional burden, the EECA needs to ensure its inclusion in and benefit from any new developments.

**Methods:**

Our 2015–16 survey aimed to collect and report on epidemiology, treatment access (including drug registration and prices, national HCV guidelines and treatment program coverage) and pertinent civil society organization (CSO) activities in 11 countries in the EECA.

**Results:**

Major gaps in epidemiological data exist; reported anti-HCV prevalence ranged from 1.5 to 7.5% for the general population, 22.7 to 70–95% for people who inject drugs (PWID) and 18 to 80% for people living with HIV (PLHIV). Ten countries (91% of the sample) have registered one or more of the second-generation, direct-acting antiviral medications (DAA) for potential interferon-free treatment. However, intellectual property issues and prices limit access to these drugs. In 2014, HCV programs in the surveyed countries covered only 0.15% of the total number of people in need of treatment. CSO-driven, international donor-funded programs are starting to fulfill needs of PWID and PLHIV.

**Conclusions:**

As feasible curative HCV treatment is now available, and given the significant regional disease burden, EECA countries need to ensure HCV surveillance and DAA availability at affordable prices in order to expand treatment and prevent the onward transmission of the infection. EECA CSOs have demonstrated their capacity to play a crucial role in advancing HCV issues, and they should continue leveraging these issues for the benefit of individual patients and public health in general.

## Background

According to a 2014 (updated in 2015, 2016 and 2017) systematic review, the 16 countries of Eastern Europe and Central Asia (EECA) are home to approximately 11.3 million people who are antibody-positive for the hepatitis C virus (HCV). HCV antibody positive (anti-HCV) prevalence estimates are 3.3% for Eastern Europe and 5.4% for Central Asia, respectively [1]. The EECA region represents almost 10% of the total global anti-HCV positive population of approximately 115 million people. Based on the estimated regional viremia prevalence of 2.5%, 6.6 million people are in need of treatment for chronic HCV. Because HCV is a blood-borne pathogen, people who inject drugs (PWID) are disproportionately affected globally. In the EECA, prevalence estimates among PWID are as high as 90% in some regional cohorts [2, 3]. HCV is known to have mutually detrimental effects with the human immunodeficiency virus (HIV) in co-infected individuals, including increased risk of death from acquired immunodeficiency syndrome and early-onset advanced liver disease. The EECA has low antiretroviral therapy (ART) coverage, estimated at 21%. This makes people living with HIV (PLHIV) in this region particularly vulnerable [4–8].

In recent years, several important developments have increased attention to viral hepatitis in general and to HCV in particular. In May 2014, the World Health Assembly, the decision-making body of the World Health Organization (WHO), passed a resolution committing WHO and its Member States to urgent action on the prevention, diagnosis and treatment of viral hepatitis. This followed the April 2014 publication of the first *WHO Guidelines for the Screening, Care and Treatment of Persons with Hepatitis C Infection*, a multi-organizational collaboration with a focus on low- and middle-income countries (LMICs) [9, 10]. Major international donors (e.g. the Global Fund, Open Society Foundations and Unitaid) are now supporting HCV programs on a much larger scale, including in EECA countries [11–13]..

Transformational change in HCV treatment is an impetus to action: new direct-acting antivirals (DAAs) allow all-oral curative protocols of significantly shorter duration. DAAs obviate the need for lengthy parenteral interferon (IFN) schedules and the extensive auxiliary resources they entail. The rapidly moving pipeline has already introduced second generation DAAs and has withdrawn first generation products. Nonetheless, DAA accessibility and costs remain major issues [14, 15]. Prices, which have been as high as 84,000 USD for a 12-week sofosbuvir course, have headlined extensive media coverage and stakeholder discussions, including United States Senate hearings [16–19].

Global efforts to address HCV diagnosis and treatment are accelerating and, given the large regional disease burden, the EECA needs to ensure its inclusion in and benefit from any new developments. EECA civil society organizations (CSOs) have already demonstrated notable achievement in the HCV arena. Greater involvement of CSOs will be a powerful force for reaching more people in need of services.

In our survey, we queried 17 CSOs in 11 EECA countries: Armenia, Azerbaijan, Belarus, Georgia, Kazakhstan, Kyrgyzstan, Moldova, Russia, Tajikistan, Ukraine and Uzbekistan. Our objective was to elucidate and report on HCV epidemiology, treatment access (including drug registration and prices, national HCV guidelines and treatment program coverage) and pertinent CSO activities. In this summary article, we report and discuss key results of this survey and propose recommendations for further action.

## Methods

Between March and April 2015, the Alliance for Public Health (Alliance, Ukraine) in collaboration with the Saint Petersburg-based ITPCru (International Treatment Preparedness Coalition) surveyed 17 CSOs in the 11 aforementioned EECA countries [20, 21]. Given the fast-changing HCV arena, the survey was repeated between March and September 2016 to update information.

Participant countries were selected between November and December 2014 on the basis of their membership on the EECA Community Advisory Board, which engages in viral hepatitis treatment access activism [22]. CSOs were chosen based on their experience in hepatitis and HIV advocacy in their respective countries. The survey instrument contained nine open-ended questions with sub-questions covering country-level HCV epidemiology among the general population, PWID and PLHIV, availability and content of HCV treatment guidelines, government and donor-funded HCV program coverage, and CSO activities. Blank tables were included for listing of HCV epidemiology data, testing, care and prevention strategies, drug registration and prices. We conducted Skype or telephone interviews with all participants to follow up on written responses, particularly regarding CSO activities.

We cross-referenced data obtained from CSOs with open-source information wherever available. We used national-source 2016 total population numbers, European Association for the Study of the Liver (EASL) and WHO HCV treatment protocols and calculated prices using June 2016 USD-local currency exchange rates listed on the www.calc.ru website unless respondents provided USD amounts. Additional patent information was obtained from WHO and the Initiative for Medicine, Access and Knowledge analyses and from CSOs involved in activities such as patent opposition (e.g. in Russia, Ukraine) [23–25]..

## Results

Between one and three CSOs in each EECA country received the questionnaire on epidemiology, treatment access (drug registration and prices, national HCV guidelines and treatment program coverage) and pertinent activities; the response rate was 17/17 (100%).

The submitted HCV prevalence data (Table 1) show a general national population anti-HCV prevalence range of 1.5 to 7.5%, with Kazakhstan and Georgia having the lowest and highest rates, respectively. Using 2016 population figures from national sources, total numbers of HCV-positive people are highest in Russia (5.9 million), Uzbekistan (1.8 million) and Ukraine (2.1 million). Prevalence among PWID ranges from 22.7% (Tajikistan) to 70–95% (Belarus). Since only small studies were available, ranges are shown for Belarus, Georgia, Moldova and Tajikistan. For PLHIV, prevalence ranges from 17.9% (Armenia) to 80% (Kyrgyzstan). PWID are specified as a key population in nine countries (82%); healthcare workers and patients undergoing invasive/hospital-level procedures are specified in three (27%).

**Table 1:** HCV prevalence data by country

Country (2016 total population)	National anti-HCV prevalence estimate (%)	Adult anti-HCV prevalence Gower, et al. (%)	Estimated number of anti-HCV+ people	Estimated anti-HCV prevalence among PWID (%)	Estimated anti-HCV prevalence among PLHIV (%)	Key populations specified
Armenia (2994400)	4		120,000	52.1	17.9	Migrants, PWID
Azerbaijan (9705600)	3.2	3.1	300,800	62.8	5.9	PWID
Belarus (9498700)	2–3	1.3	250,000	70–95		PWID
Georgia (3720400)	7.5		208,800	57–74	4.8	PWID, MSM, healthcare workers
Kazakhstan (17753200)	1.5–3	3.3	255,000–510,000	% not available (estimated 6049 people)	4.5	
Kyrgyzstan (6019500)	4	2.5	220,000	45.2	80	
Moldova (3553100)	1.7–4	4.5	60,000–142,000	35.3–65.4	4.6	PWID
Russia (146544710)	4	4.1	5,900,000	69	27	PWID
Tajikistan (8547000)	2.3	3.1	200,000	22.7–49.3	25.6% among HIV+ PWID	PWID
Ukraine (42708647)	5		2,135,000	55		PWID; hemophiliacs, hemodialysis patients; MSM; PMTCT clients
Uzbekistan (31575300)	6.5		1,800,000	36		Healthcare workers, PWID, patients receiving invasive treatment

With respect to treatment access, one or more second generation DAAs are registered in 10 (91%) of 11 countries (Table 2). This includes generic sofosbuvir in six countries, combination sofosbuvir/ledipasvir in three countries and combination ombitasvir/paritaprevir/ritonavir/dasabuvir in six. For daclatasvir, the original product is registered in Russia; generic registration is pending in Kyrgyzstan; and generic manufacture and sale without royalty payments will occur in Azerbaijan, Georgia and Ukraine under an agreement between Bristol-Myers Squibb and the Medicines Patent Pool. Simeprevir is registered in five countries and available through a humanitarian aid program in Uzbekistan.

**Table 2:** Direct-acting antiviral drug registration in surveyed countries (September 2016)

Country	sofosbuvir	sofosbuvir generic	sofosbuvir/ ledipasvir	sofosbuvir/ ledipasvir generic	daclatasvir	daclatasvir generic	ombitasvir/ paritaprevir/ ritonavir/ dasabuvir (Viekira Pak)	ombitasvir/ paritaprevir/ ritonavir	dasabuvir	simeprevir	asunaprevir	narlaprevir	boceprivir	telaprevir
Armenia													Yes	Yes
Azerbaijan		Yes				pending	Yes						Yes	Yes
Belarus							Yes						Yes	Yes
Georgia	Yes		Yes			pending							Yes	Yes
Kazakhstan			pending				Yes			Yes			Yes	Yes
Kyrgyzstan		Yes	Yes	Yes		pending								
Moldova		Yes						Yes	Yes	Yes			Yes	Yes
Russia	Yes				Yes		Yes			Yes	Yes	Yes	Yes	Yes
Tajikistan		Yes												
Ukraine	Yes	Yes	Yes			pending	Yes	Yes	Yes	Yes			Yes	Yes
Uzbekistan		Yes								humanitarian			Yes	

The first generation DAAs boceprivir and telaprivir, which are no longer recommended, are registered in nine (82%) and eight (73%) countries, respectively. Additionally, older pegylated interferon (PEG-IFN) original and/or biosimilar products are available in all countries.

Reported DAA prices vary among countries and procurement sources (public and private). Twelve-week generic sofosbuvir treatment price ranges in cost from USD 780 in Kyrgyzstan to USD 2805 in Ukraine; donor-supported supported programs lead by the Government in Georgia and run by ICF "Alliance for Public Health" in Ukraine are simultaneously providing it free of charge. Combination ombitasvir/paritaprevir/ritonavir/dasabuvir costs USD 16,749 per 12-week course in Belarus. A 12-week course of originator simeprevir ranges from USD 9840 (Russia) to USD 25,407 (Moldova). Armenia, Belarus, Kazakhstan, Kyrgyzstan, Russia, Ukraine and Uzbekistan reported “buyers’ clubs” that arrange “personal-use” importation of primarily Indian and Egyptian generic sofosbuvir, sofosbuvir/ledipasvir and daclatasvir for their members. Prices for sofosbuvir obtained through the "buyers’ club" range from 465 USD per 12-week course in Belarus and Ukraine (versus USD 2805 for registered generic in the latter) and USD 390/12-week course in Uzbekistan (versus USD 825 for registered generic). Table 3 shows lowest available September 2016 prices by country.

**Table 3:** Lowest direct-acting antiviral drug prices in surveyed countries - USD per 12-week course (September 2016)

Country	sofosbuvir generic	sofosbuvir generic ‘buyers’ club’	ombitasvir/ paritaprevir/ dasabuvir/ ritonavir	simeprevir	daclatasvir	asunaprevir
Azerbaijan	1011		15,000	7200		
Belarus		465	16,749			
Kyrgyzstan	780					
Russia		360	13,770	9840	5556	558
Ukraine	2805	465				
Uzbekistan	825	390				

PEG-IFN is still used for treatment of HCV in some countries, prices range from USD 3038 to USD 13,300 for a 48-week course, with ribavirin ranging from USD 250 to 3500.

Additional activities to improve treatment access were reported: Sofosbuvir patent opposition is in progress in Russia and Ukraine. Kyrgyzstan has amended its laws on intellectual property to better accommodate trade-related aspects of intellectual property rights (TRIPS). DAA patents have been issued for daclatasvir and simprevir by the Eurasian Patent Organization (EAPO), which includes Armenia, Azerbaijan, Belarus, Kazakhstan, Kyrgyzstan, Russia, Tajikistan and Turkmenistan; for ABT-450, daclatasvir, simeprevir and sofosbuvir by Russia; and for ombitasvir by Ukraine.

HCV treatment guidelines are available in nine countries (82%). Armenia has an adapted WHO version of guidelines for only HCV-HIV co-infection, and Tajikistan reported using the Russian guidelines. PEG-IFN is listed as a first-line medication in all existing EECA guidelines. Kazakhstan, Moldova, Russia and Ukraine also include first-generation DAAs that are no longer recommended. Georgia added sofosbuvir with the launch of its donor-funded program in 2015 and is in the process of updating its national guidelines, as are Azerbaijan and Belarus.

Regarding access to HCV treatment services, in 2014, programs covered a regional total of 10,000 people (0.15% of the total 6.6 million estimated in need of treatment), mainly in Kazakhstan and Russia, using IFN-based regimens. In 2015, donor-funded programs using combination DAA-IFN protocols were expected to cover 5000 people in Georgia and 1500 in Ukraine. Gilead Sciences, Inc. (developer of sofosbuvir) reportedly plans future expansion to provide 20,000 treatment courses per year in Georgia [26].

CSO activities included HCV awareness raising, mobilization, advocacy, testing and treatment to promote patient rights and appropriate policy and service provision. Formal evaluation/quantitative results were generally not available. Activities are categorized and highlighted in Tables 4 and 5.

**Table 4:** Civil society organization HCV activity summary

Category	Activities			
Awareness raising	National campaigns to inform decision makers and the general public	Patient ‘schools’ on access to treatment and other HCV issues	Media notices on HCV	Educational videos
Mobilization	Establishing networks of individuals and organizations to advocate HCV issues	Implementing letter writing/petition signing campaigns, e.g. a ‘Treatment Waiting List’ and lower price requests to pharmaceutical companies	Organizing research and reporting on HCV needs	Organizing CSOs to meet with pharmaceutical companies
Advocacy	Encouraging national treatment programs and guideline development	Organizing campaigns aiming to bring about favorable policy and response from governments, industry and donors	Negotiating lower prices with pharmaceutical companies	Specific opposition to pharmaceutical patent applications and support for alternate licensing
Testing and treatment	Conducting screening test campaigns to identify HCV- antibody positive people (that also raise awareness and provide evidence for advocacy)	Implementing treatment programs integrated into existing services, particularly for the disproportionately affected/marginalized, e.g. PWID	Seeking donor funding for expanded treatment programs using updated protocols	Providing community-level and technical input on national program planning and guideline committees

EECA Civil Society Involvement in HCV Work

**Table 5:** Selected civil society organization HCV activity examples

Category	Activity	Organization(s) involved	Results and/or comments
Awareness raising	National campaign using mass media and celebrities in Georgia	Concerted effort by Georgian Harm Reduction Network, OSF Georgia, Georgia CAB, Medecins du Monde, Hepa+, New Vector and others	Large-scale treatment program implemented
	“Demand treatment!” campaign in Ukraine	Concerted effort of 90 CSOs led by Alliance for Public Health	Community-level HCV screening to document prevalence; subsequent national and regionally/locally-funded HCV treatment programs
Mobilization	HCV advocacy and policy brief research and dissemination in 11 countries	Eurasian Harm Reduction Network; Kyrgyz Harm Reduction Network; Andrey Rylkov Foundation (Russia; Treatment Preparedness Coalition (Russia); Alliance for Public Health	Evidence compiled for awareness-raising and advocacy activities
	Drug registration, pricing, clinical trial and early access issue negotiation at EECA level	EECACommunity Adivsory Board meetings addressing AbbVie, BMS, Gilead, Janssen and Merck Sharp & Dohme	Inclusion of simeprevir on the Russian “List of Vital and Essential Medicines” and removal of telapravir, which was included due to industry price reduction (market segmentation) despite being obsolete
Advocacy	DAA price reduction negotiation in Ukraine	Alliance for Public Health	USD 750 for a 12-week sofosbuvir course; USD 900 for a 12-week sofosbuvir/ledipasvir course
	National treatment guideline modification and program endorsement advocacy neogtiations		Inclusion of DAAs in national treatment guidelines and establishment of a national and over 15 regional treatment programs; government procurement of sofosbuvir
Testing and treatment	HCV treatment for HIV-HCV coinfected PWID implementation in harm reduction programs in Ukraine	Alliance for Public Health	Global Fund support and DAAprice reduction (above) obtained
	Contribution to national HCV guideline development/revision and strategic planning	Multiple CSOs inAzerbaijan, Georgia, Kazakhstan, Kyrgyzstan, Moldova and Ukraine	Supports appropriate policy and program implementation

## Discussion

Chronic untreated HCV infection progresses to conditions such as cirrhosis and hepatocellular carcinoma, which eventually lead to death. Despite an overall decline in new infection incidence globally, mortality due to chronic HCV is still high and may increase in coming years due to existing high infection rates and lack of connection to diagnosis and treatment [27, 28]. However, with treatment, chronic HCV infection is curable [29, 30]. Different combinations of available DAAs allow oral, IFN-free treatment for different HCV genotypes, while the new sofosbuvir/velpatasvir is pan-genotype [31, 32]. DAAs, especially broad coverage/pan-genotype combinations, simplify chronic HCV infection diagnosis and treatment such that it can be delivered through a range of service platforms to increase accessibility for PWID, PLHIV and the general population in comparison to complicated IFN-based protocols. DAA treatment has also been shown to have a reduced cost when compared to long-term treatment of chronic HCV infection complications [33, 34]. Thus, access to DAAs is particularly important for EECA countries with high HCV burdens. Our survey has identified specific information gaps as well as valuable knowledge, which can be utilized in EECA countries to advance DAA-based HCV treatment towards national and global hepatitis control objectives.

In the 11 countries we surveyed, HCV epidemiological data that is required to define response and plan services, including commodities procurement, are lacking. Anti-HCV prevalence data are more often available while HCV viremia confirmation data, as well as details on genotyping and staging, are less forthcoming. Georgia is an exception with its comprehensive prevalence surveys and viremia confirmation testing protocols. Overall, the data submitted by CSOs in this survey are consistent with cited regional estimates, i.e. anti-HCV prevalence in Eastern Europe is 3.3% (range 1.6–4.5%) and is 5.4 (range 3.5–6.8%) in Central Asia. The infection prevalence rates among PWID and PLHIV are, as expected, consistently higher than general population rates [1]. Survey data support the hypothesis that initial decision-making based on published estimates is a reasonable starting point to fill epidemiological information gaps.

All surveyed countries need to address DAA registration and pricing promptly in order to implement HCV treatment per current international guidelines. The rapid pace at which products are introduced leaves registration lagging. At the same time, pharmaceutical company maneuvers, such as market segmentation with telaprevir and simprevir in Russia (Table 5), have caused further delay in the establishment of affordable optimal treatment protocols. Patented products and their associated high prices also act as barriers to access as exemplified in Russia, where sofosbuvir patent opposition is in progress and only buyers’ club supplies are available at this time. Countries must negotiate with manufacturers for voluntary licensing agreements (which the Medicines Patent Pool can facilitate) and the key TRIPS flexibility of compulsory licensing as needed to facilitate generic production, which has been projected to cost as little as USD 100–250 per 12-week DAA treatment course with two or three drug combinations [35–37].

There has been progress in the past year based on the first and second rounds of our survey, with more countries registering second generation DAAs. Notably, the majority now have an IFN-free DAA regimen option for most HCV genotypes. Several surveyed countries also achieved increased availability of generic DAAs and lower prices, albeit some through personal use imports via  buyers’ clubs. Generic daclatasvir manufacture in several EECA countries has been agreed upon with Medicines Patent Pool involvement. In Ukraine, successful negotiations with manufacturers yielded prices much closer to projected targets – sofosbuvir is USD 750 and sofosbuvir/ledipasvir is USD 900 per 12-week course (Table 5).

A survey of HCV guidelines in the participating countries indicated that there are generally guidelines for first-line treatment with IFN- and/or first generation DAA-based protocols. WHO, EASL and the American Association for the Study of Liver Diseases all recommend second-generation oral DAA-based regimens [10, 23, 38]. Up-to-date national guidelines will underpin policy and program development and anticipate appropriate drug registration and supply. Countries should seek technical support as needed to stay up-to-date with current knowledge; for example, CSOs have recently been an important source of expertise in Azerbaijan, Georgia, Kazakhstan, Kyrgyzstan, Moldova and Ukraine.

Treatment programs in the surveyed countries have had minimal coverage rates, i.e. a total of 0.15% of the population in need in 2014. Donor-supported programs in Georgia and Ukraine are introducing second-generation DAAs. While these services currently target the disproportionately affected, they can also serve as examples for healthcare workers, hospital-treated patients and other groups who are at risk for iatrogenic HCV transmission in settings of suboptimal infection control such as those of the EECA and other LMIC healthcare systems. Substantial treatment program scale-up through domestic and international collaboration is needed. Implementation partners who can expand their services by integration of HCV care into existing platforms can fulfill priority needs with the added advantage of established knowledge of and acceptance from “hard-to-reach” and high-risk people, as seen in Ukrainian harm reduction programs.

CSOs in the surveyed countries have executed awareness raising and advocacy campaigns that have gathered HCV data and improved treatment access in terms of increased DAA availability, reduced medication prices, updated guidelines and expanded services. EECA CSOs should also capitalize on global experience with HIV ART patent opposition and price reduction that has informed HCV activities in Brazil, Egypt, the European Union and India, where as a result, one DAA patent has been revoked. Based on survey information, recommendations for further CSO action are listed in Table 6.

**Table 6:** Recommendations for stakeholders

Based on this survey, we recommend that CSOs:
• conduct HCV epidemiology studies in key populations;
• integrate HCV services in PWID and PLHIV programs;
• monitor PWID access to HCV treatment; document, disseminate and, if appropriate, pursue legal and media options for any cases of service denial;
• advocate for inclusion of PWID in national HCV guidelines and programs;
• advocate for HCV medication price reductions through stakeholders and the media;
• monitor the drug registration landscape and maintain pressure on pharmaceutical companies and governments for prompt registration of new drugs;
• partner with international organizations to oppose barriers and leverage TRIPS flexibilities;
• work with governments on regulatory measures to ensure lower prices for generics;
• advocate for government disclosure of prices, purchase volumes and relevant treatment program details to allow independent expert evaluation;
• participate on national HCV program advisory committees;
• participate in advocacy related to keeping WHO guidelines updated;
• include HCV testing and treatment in project proposals, with focus on key populations but also considering the general population;
• document and disseminate CSO-led HCV program best practices.

## Conclusions

Highly effective curative HCV treatment is now available, and given the significant regional burden, EECA countries need to establish epidemiologic surveillance and initiate appropriate response, including ensuring DAA availability at affordable prices in order to implement programs with updated treatment guidelines and expanded coverage, particularly for PWID and PLHIV.

Eastern European and Central Asian Civil Society Organizations have demonstrated capacity to play a crucial role in advancing HCV issues, which they should continue leveraging for individual patient and global health benefit.

## Abbreviations

ABT-450: Paritaprevir
anti-HCV: HCV antibody positive
ART: Antiretroviral therapy
CSO: Civil society organization
DAA(s): Direct-acting antiviral medication(s)
EAPO: Eurasian patent organization
EASL: European Association for the Study of the Liver
EECA: Eastern Europe and Central Asia
HCV: Hepatitis C virus
HIV: Human immunodeficiency virus
IFN-free: Treatment regimen without interferon
LMIC: Low- and middle-income countries
PEG-IFN: Pegylated interferon
PLHIV: People living with HIV
PWID: People who inject drugs
TRIPS: Agreement on trade-related aspects of intellectual property rights
WHO: World Health Organization
